# Drugs Interfering with Insulin Resistance and Their Influence on the Associated Hypermetabolic State in Severe Burns: A Narrative Review

**DOI:** 10.3390/ijms22189782

**Published:** 2021-09-10

**Authors:** Maria Greabu, Silviu Constantin Badoiu, Iulia-Ioana Stanescu-Spinu, Daniela Miricescu, Alexandra Ripszky Totan, Silvia Elena Badoiu, Michel Costagliola, Viorel Jinga

**Affiliations:** 1 Department of Biochemistry, Faculty of Dental Medicine, Carol Davila University of Medicine and Pharmacy, 8 Eroii Sanitari Blvd, 050474 Bucharest, Romania; maria.greabu@umfcd.ro (M.G.); daniela.miricescu@umfcd.ro (D.M.); alexandra.totan@umfcd.ro (A.R.T.); 2 Department of Anatomy and Embriology, Faculty of Medicine, Carol Davila University of Medicine and Pharmacy, 8 Eroii Sanitari Blvd, 050474 Bucharest, Romania; 3 Department of Plastic and Reconstructive Surgery, Life Memorial Hospital, 365 Grivitei Street, 010719 Bucharest, Romania; 4 Faculty of Medicine, Carol Davila University of Medicine and Pharmacy, 8 Eroii Sanitari Blvd, 050474 Bucharest, Romania; silvia.badoiu@stud.umfcd.ro; 5 Department of Plastic and Reconstructive Surgery, Faculté de Médicine Toulouse-Rangueil 3, Université Toulouse III-Paul Sabatier, Rue de Languedoc, CEDEX 04, 31000 Toulouse, France; costagliola.m@wanadoo.fr; 6 Department of Urology–Clinical Hospital Prof. Dr. Th. Burghele, Faculty of Medicine, Carol Davila University of Medicine and Pharmacy, 37 Dionisie Lupu Street, 030167 Bucharest, Romania; viorel.jinga@umfcd.ro

**Keywords:** burns, insulin resistance, insulin signaling pathway, β blockers, statins, thiazolidinediones, metformin

## Abstract

It has become widely accepted that insulin resistance and glucose hypermetabolism can be linked to acute pathologies, such as burn injury, severe trauma, or sepsis. Severe burns can determine a significant increase in catabolism, having an important effect on glucose metabolism and on muscle protein metabolism. It is imperative to acknowledge that these alterations can lead to increased mortality through organ failure, even when the patients survive the initial trauma caused by the burn. By limiting the peripheral use of glucose with consequent hyperglycemia, insulin resistance determines compensatory increased levels of insulin in plasma. However, the significant alterations in cellular metabolism lead to a lack of response to insulin’s anabolic functions, as well as to a decrease in its cytoprotective role. In the end, via pathological insulin signaling associated with increased liver gluconeogenesis, elevated levels of glucose are detected in the blood. Several cellular mechanisms have been incriminated in the development of insulin resistance in burns. In this context, the main aim of this review article is to summarize some of the drugs that might interfere with insulin resistance in burns, taking into consideration that such an approach can significantly improve the prognosis of the burned patient.

## 1. Burns and Insulin Resistance: Background of a Toxic Relationship

Burn injury represents a major form of trauma, not only impacting the skin, but also producing disturbances to the underlying and distant tissues. In severe cases, local inflammation is associated with a general inflammatory response that reaches all the body systems. Most commonly determined by a thermal source, burns can also be produced by electricity or chemicals. In very rare situations, patients suffer burn injuries following ionizing radiation. Although in the last decades significant advances have been made in therapy, burn wounds continue to be life-threatening. The inflammatory process associated with the burn induces significant plasma leakage into the interstitial fluid and can lead to multiple organ dysfunction, thus promoting a further deterioration of the patient, sustained by the following persistent hypermetabolic state. Furthermore, this phase is characterized by a reduced healing rate of the wound and a diminishment of cell-mediated immunity [1].

Even for patients who survive the initial critical hours after the burn injury, the death risk is still increased, mostly because of subsequent infections [2]. Additionally, patients with respiratory complications produced through inhalation of smoke (which contains carbon monoxide, hydrogen cyanide (HCN), sulfur dioxide, hydrogen sulfide (H_2_S), phosgene, ammonia, formaldehyde, etc.) [3] present an increased risk [4]. Moreover, local inflammation caused by the discharge of high levels of cytokines, hypermetabolic state, insulin resistance, and muscle wasting are all hallmark traits of the clinical presentation of a severely burned patient (where the burn injury involves more than 40% of the total body surface area (TBSA)) and represent major complications in treatment [1,2].

The profound consequences that severe burns have on patients promote high morbidity and mortality, with over 330,000 people dying as a result each year [5]. As previously mentioned, one of the main contributors to such an increased number of yearly deaths is represented by the associated insulin resistance [6]. Insulin resistance associated with hyperglycemia has been linked to a higher risk of infection (pneumonia and bloodstream infections), deficient wound healing, increased catabolism in the muscle, and significant loss of skin grafts [7].

Produced by the β cells of the pancreas, insulin contains 51 amino acids distributed in two chains (A-21 amino acids and B-30 amino acids) connected by disulphide bridges. The primary role of insulin is to maintain normal glucose levels in the blood. It plays an anabolic role in all metabolisms (carbohydrate, lipid, and protein), enables glucose uptake in the cell, and has mitogenic effects [8].

In order to fulfil its roles, insulin binds to a specific insulin receptor (IR), a heterotetramer situated on the cell membrane and formed by four glycoprotein chains (2α and 2β) connected by disulphide bonds. The binding of insulin to the α subunit of the IR determines a conformational shift that allows ATP to attach to the β subunit, thus promoting its phosphorylation, stimulating tyrosine kinase activity, which further stimulates Tyr (tyrosine) phosphorylation of IRS (insulin-responsive substrates). These intracellular proteins subsequently attach to new signaling molecules that mediate insulin’s further cellular activities [8,9].

After insulin binds to the insulin receptor (IR), the IRS/PI3K/AKT and MEK/ERK (Ras/mitogen-activated protein kinase kinase/extracellular signal-regulated kinase) signaling pathways are activated [10]. The phosphoinositide 3-kinase (PI3K) signaling pathway is involved in the regulation of very important metabolic events, including the synthesis of glycogen, proteins, and lipids, and it controls glucose transporter protein translocation and gluconeogenesis in the liver, while insulin’s anabolic, mitogenic, and pro-hypertrophic actions are all linked to the MAPK (mitogen-activated protein kinase) pathway [11]. Following the Tyr phosphorylation of the β subunits, PI3K attaches to the receptor complex. Subsequently, AKT/PKB (protein kinase B) is phosphorylated and, thus, activated [12,13]. AKT is an intracellular enzyme responsible for glucose uptake and glycogen synthesis; AKT in skeletal muscle is activated by insulin, but, in burns, this insulin-mediated activation is impaired. This results in muscle wasting and metabolism alterations [14], which are characteristic of severe burns.

Through the phosphorylation of AKT, PI3K is considered to have key functions in controlling insulin signaling metabolic effects, promoting glucose transport. Additionally, this signaling cascade can also influence the localization of GLUT4 (glucose transporter 4) on the plasma membrane [6]. Glucose peripheral use and energy storage are promoted by this membrane protein, which, in skeletal muscles and adipose tissue, acts as the main insulin-responsive glucose transporter and controls glucose transfer into the cells [15]. Moreover, previous research has revealed that AKT is also involved in maintaining glucose homeostasis in the liver, inhibiting hepatic gluconeogenesis and glycogenolysis, and influencing several quintessential enzymes implicated in glycolysis [6].

SH2 (Src homology 2) domain proteins, such as the regulatory subunits of class 1A PI3K, GRB2/SOS (growth factor receptor-bound protein 2/son of sevenless), and SHP2 (SH2 domain containing protein tyrosine phosphatase-2), are activated by Tyr phosphorylation of IRS [16]. Subsequently, PIP3 (phosphatidylinositol 3,4,5-trisphosphate) is produced in the plasma membrane through the phosphorylation of PIP2 (phosphatidylinositol 4,5-bisphosphate) by PI3K and, in turn, recruits AKT, which is then activated through the phosphorylation of the T308 (threonine 308) site of protein kinase B and other analogues in aPKCs λ/ι and ζ (calcium- and diacylglycerol-independent atypical protein kinase C isoforms λ/ι and ζ) [17,18].

Previous research has shown that the activation of the PI3K and AKT signaling pathways triggers upregulation of pro-inflammatory cytokines, including tumor necrosis factor-α (TNF-α) and interleukin 6 (IL-6). On the other hand, insulin inhibits the activity of GSK3β (glycogen synthase kinase-3beta), thus sustaining the hypothesis regarding insulin’s protective role against systemic inflammation and preventing organ dysfunction. Moreover, in its attempt to attenuate the inflammatory process, insulin mediates the functions of several transcription factors or molecules (MKP-1—mitogen-activated protein kinase phosphatase-1; STAT3 or 5—signal transducer and activator of transcription 3 or 5; NFκB—nuclear factor kappa-light-chain-enhancer of activated B cell; SOCS-3—suppressor of cytokine signaling-3) [19].

Insulin resistance is defined as the tissues’ diminished response to insulin’s effects. While insulin resistance is characteristic of chronic metabolic disorders, it was Claude Bernard in 1877 who observed and portrayed hyperglycemia as a consequence of an acute stress, such as a hemorrhagic shock, affirming for the first time that it is not only diabetic patients who can experience it. Since then, it has become widely accepted that insulin resistance and glucose hypermetabolism can be linked to acute pathologies, such as burn injury, severe trauma, or sepsis [20,21]. Severe burns can determine a significant increase in catabolism, having an important effect on glucose metabolism and on muscle protein metabolism. It is imperative to acknowledge that these alterations can lead to increased mortality through organ failure, even when the patients can survive the initial trauma caused by the burn [10].

Unlike insulin resistance in metabolic disorders, which develops over the course of months or even years, in severely burned patients, this newly acquired condition has a fast onset, representing an acute complication, caused by the activation of several signaling pathways that impair insulin receptors through a high level of stress hormones, overproduction of pro-inflammatory cytokines, or mitochondrial oxidative stress [6,10,22].

The insulin–insulin receptor axis has proven to be crucial for glucose metabolism homeostasis and in critical conditions such as burn trauma, where stress hyperglycemia initially plays a redeeming role. Peripheral insulin resistance is hallmarked by hyperglycemia associated with compensatory hyperinsulinemia (by augmented pancreatic insulin production). So, by limiting the peripheral use of glucose, insulin resistance determines increased levels of insulin in plasma. However, the significant alterations in cellular metabolism lead to a lack of response to insulin’s anabolic functions, as well as to a decrease in its cytoprotective role. In the end, via pathological insulin signaling associated with increased liver gluconeogenesis, elevated levels of glucose circulate in the blood [22].

**Figure 1:**
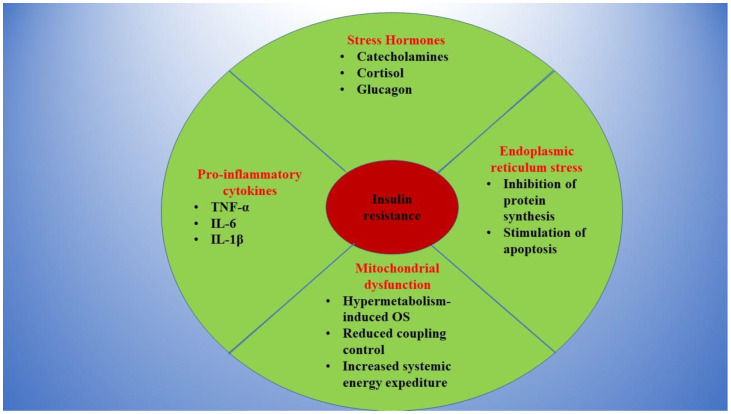
The main contributors to insulin resistance in burns.

Several cellular mechanisms have been incriminated in the development of insulin resistance in burns (Figure 1), including an increased level of FFAs (free fatty acids) or elevated levels of SOCS (suppressors of cytokines signaling) proteins, leading to a reduction in IRS signaling [10].

Moreover, the activation of the two aforementioned pathways can also be produced by oxidative stress. Thus, overproduction of ROS (reactive oxygen species) or an increased level of stress in the endoplasmic reticulum will impair insulin signaling as well. Furthermore, an upsurge in the activity of the phosphatase tensin homolog (PTEN) or Srchomology 2 domain containing inositol 5′-phosphatase (SHIP) may determine a downturn in insulin signaling, thereby contributing to insulin resistance [10].

Alongside MKP-1, hormones with antagonist effects on insulin, such as catecholamines or glucagon, and muscle wasting can also contribute to insulin resistance. Previous research performed on animal models showed that insulin resistance can be caused by shortcomings in IR activation or lack of activation of the signaling pathway by insulin binding to the IR [14].

Moreover, Jeschke et al. showed that insulin receptor signaling can be impaired by increased endoplasmic reticulum stress (ERS), thus contributing to insulin resistance alongside mitochondrial dysfunction. Stress mediators and pro-inflammatory cytokines may increase ERS/unfolded protein response (UPR), affecting the PI3K/AKT signaling pathway and promoting insulin resistance and hyperglycemia. ERS and UPR are considered to be intracellular signaling pathways linked to the inflammatory process [23].

Severe burns cause significant metabolic alterations, determining physiological stress that promotes hyperglycemia through increased levels of catecholamines, glucagon, and corticosteroids. The release of inflammatory mediators, such as cytokines TNF-α, IL-6, and MCP-1 (monocyte chemotactic protein 1), alters insulin resistance in skeletal muscles. Hence, alongside the stimulation of glycogenolysis by catecholamines, these pro-inflammatory factors contribute to a hypermetabolic state characterized by hyperglycemia. Furthermore, at the same time, peripheral insulin resistance occurs through blockage of the insulin signaling pathway and GLUT4 [7].

In this context, the main aim of this review article is to summarize some of the drugs that might interfere with insulin resistance in burns, taking into consideration that this treatment approach can significantly improve the prognosis of the burned patient by using medications that decrease insulin resistance and by discarding drugs that augment insulin resistance and accentuate the hypermetabolic state (which is typical in severe burns).

## 2. Drugs That Might Interfere with Insulin Resistance in Burns

Insulin resistance and glucose control are obviously affected not only by medication, but also by other factors, including: diet, age of the burned patient, genetics, and the presence of other injuries or other comorbidities besides severe burns. Moreover, previous medication administered to the patient must be taken into consideration, as these drugs might influence their metabolic responses. Therefore, it is essential for the physician to be informed on these aspects and include them in the therapeutical approach.

Some of the drugs that we present here are used in the treatment of pathological conditions that may affect the patient before sustaining the burn trauma, such as:

- β blockers, angiotensin-converting enzyme inhibitors, and angiotensin II receptor blockers for the treatment of cardio-vascular conditions;
- Antipsychotic drugs for the treatment of psychiatric conditions;
- Vitamin B3 and statins for the treatment of dyslipidemias;
- Thiazolidinediones and metformin for the treatment of type II diabetes mellitus.

Nevertheless, there are situations when such drugs (β blockers, antipsychotic drugs, thiazolidinediones, metformin) are used in the treatment of burn trauma “per se” or in the treatment of the complications of burn trauma.

It is of utmost importance to know which drugs may increase insulin resistance (and thus need to be avoided) and which drugs decrease insulin resistance and might be considered for administration in a severely burned patient in a hypercatabolic–hyperglycemic state (characterized by insulin resistance and systemic inflammatory response). The inclusion or exclusion of any drugs from the treatment of a burned patient cannot be made mechanistically, based only on theoretical extrapolations. It is crucial to clarify that the clinical and biochemical image (of the patient) primarily dictate the course of action and treatment in a hospital setting.

### 2.1. β Blockers

β blockers represent a class of drugs commonly used in the treatment of burned patients, with good results [24,25,26,27]. The blockade upon the adrenergic receptors is justified by the fact that catecholamines are major inducers of the hyperdynamic syndrome [28,29], hypercatabolic state [28], and systemic inflammatory response [29] in severe burns.

The elevated levels of circulating catecholamines initiate the acute stress response in severe burns, which is further amplified and sustained by cytokines [30]. The persistency of increased levels of catecholamines for months or years has a deleterious effect upon the severely burned patient [31].

**Figure 2:**
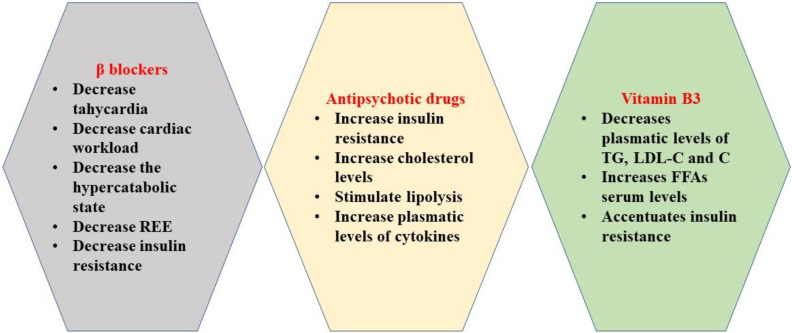
Effects of β blockers, antipsychotic drugs, and vitamin B3.

Besides their beneficial results on the cardiovascular system, decreasing tachycardia and reducing the cardiac workload (Figure 2) [24], β blockers have important metabolic effects, contributing to a global reduction of the hypercatabolic state through multiple mechanisms [25] that address glucose, fat, and protein metabolism.

Regarding insulin resistance in burned patients, previous studies have affirmed an improvement in insulin sensitivity as an indirect effect of β blocker administration, via the reduction of ERS [26,32] and the mitigation of UPR [32]. Other studies performed on burned patients reported a diminishment of insulin resistance together with a decrease in the rate of energy expenditure (REE) and lipolysis [25] or a decline in insulin resistance associated with a reduction in REE and an increase in peripheral lean mass [33].

On the other hand, studies have been conducted on patients with arterial hypertension with or without diabetes mellitus or metabolic syndrome to assess the effects of non-selective β blockers (propranolol, atenolol, metoprolol) and selective β1 blockers or vasodilating β blockers (dilevalol, carvedilol, celiprolol, nebivolol) on insulin sensitivity. Most of these studies reported that non-selective β blockers decrease insulin sensitivity [34,35] and worsen serum lipid profile abnormalities [35,36,37], while vasodilating β blockers increase insulin sensitivity and ameliorate the plasmatic lipid profile [35,36,37,38]. Therefore, the usage of vasodilating β blockers might be preferable [35,36,37,38,39].

### 2.2. Antipsychotic Drugs

Psychiatric evaluation in the acute phase of burns was reported to be necessary in almost one-quarter of the patients admitted in a regional burns center [40], but it is considered that the proportion of burned patients in need of appropriate psychiatric care is much larger, in both the acute and chronic phases [41,42,43].

The prevalence rates of discovered psychiatric disorders or substance abuse exceeded 40% and 50%, respectively, in the acute phase of the trauma [40]. Roughly, the cause for psychiatric consultation in the acute phase was represented in almost half of the cases by a psychiatric disorder (agitation, generalized anxiety, acute stress reaction, depression, delirium, inability to cope with the situation), in about one-third of the patients by pain control, and in approximatively one-fifth of the patients by alcohol abuse [40]. In total, 91.5% of the consulted patients needed psychiatric medication [40].

Some of the prescribed drugs, especially the antipsychotic medications, are known for their secondary metabolic effects. It was proved that quetiapine, olanzapine, and clozapine induce hypercholesterolemia [44] and increase insulin resistance, determining augmented lipolysis and elevated serum levels of free fatty acids and triglycerides [45]. It was reported that clozapine, olanzapine, quetiapine, and risperidone induce insulin resistance, cause obesity, and amplify the risk of type II diabetes mellitus in patients with otherwise normal tolerance for glucose [46,47].

Clozapine induces insulin resistance [48], abdominal obesity [49], arterial hypertension [50], dyslipidemia [50,51], and hyperglycemia [52,53] and increases the plasmatic levels of catecholamines [54,55]. Additionally, several animal studies demonstrated beyond doubt that clozapine and olanzapine induce insulin resistance, with important metabolic consequences on glucose, fat, and protein metabolism [41,56,57].

Different mechanisms were proposed for the induction of insulin resistance by second-generation antipsychotic drugs [41,58]. It seems that olanzapine induces insulin resistance mediated by the IRS/PI3K/AKT signaling pathway [41]. Insulin resistance and its consequences become obvious after 6–8 weeks of treatment [41,56,57,58]. Taking into consideration the large number of severely burned patients requiring long-term psychiatric treatment, the problem of drug-induced insulin resistance cannot be neglected.

### 2.3. Vitamin B3 (Niacin)

Niacin is essential for the production of NAD (nicotinamide adenine dinucleotide) and NADP (NAD phosphate), which are necessary for cell energy production through oxidative phosphorylation [59,60,61,62]. It has been commonly used for the treatment of dyslipidemias, especially for hypertriglyceridemia and elevated LDL-C (low-density lipoprotein-cholesterol) [62,63]. Niacin decreases plasmatic levels of triglycerides (TG), LDL-C, and total cholesterol (C) [62].

Chronic treatment with niacin induces insulin resistance through its downregulatory effect on genes involved in the insulin signaling pathway [64]. It also induces an increase in FFA serum levels, which further accentuates the insulin resistance [65]. Therefore, the administration of this drug in burned patients that have been previously treated with niacin for dyslipidemia requires caution.

### 2.4. Angiotensin-Converting Enzyme Inhibitors (ACEI)

Angiotensin-converting enzyme inhibitors (captopril, enalapril, lisinopril, benazepril, fosinopril, perindopril, ramipril, etc.) are currently used in the treatment of arterial hypertension [66], heart failure [67], post myocardial infarction [67,68], asymptomatic left ventricular dysfunction [69], chronic kidney disease [70], etc.

**Figure 3:**
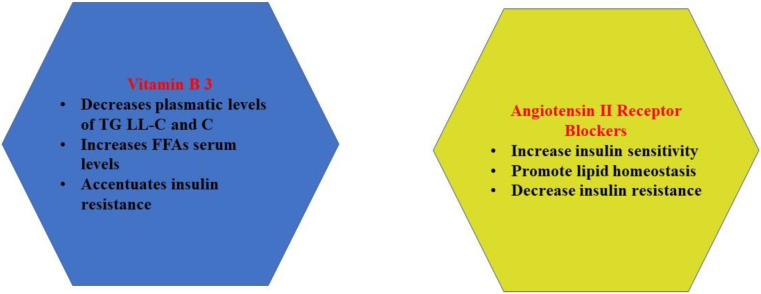
Effects of ACEI and ARBs (Angiotensin II Receptor Blockers).

Their pharmacologic action consists of inhibiting the conversion of angiotensin I to angiotensin II, with a subsequent decrease in blood pressure (Figure 3) through multiple mechanisms: lack of inactivation of bradykinin, resulting in peripheral vasodilation [71], decreased sympathetic tone in the central nervous system, diminished release of catecholamines from the adrenal medulla, etc. Another consequence of inhibited conversion of angiotensin I to angiotensin II is increased natriuresis [71]. ACEI also have cardioprotective effects by increasing the circulating levels of N-acetyl-seryl-aspartyl-lysyl-proline (Ac-SDKP), a natural inhibitor of pluripotent hematopoietic stem cell proliferation [72].

One of the “secondary” effects of ACEI is an amplification of insulin sensitivity and glucose tolerance in patients with insulin resistance [73,74] in such an efficient manner that some ACEI were proven to prevent the development of type 2 diabetes mellitus [75].

Moreover, in diabetic mice, Temocapril improves insulin resistance through increasing glucose uptake in skeletal muscle, due to the activation of the bradykinin–nitric oxide (NO) system and a consequent increase in translocation of GLUT4 [76].

The amelioration of insulin resistance in humans by the administration of ACEI has been demonstrated through several clinical studies [73,74], but the molecular mechanism remains to be elucidated. Nevertheless, the use of ACEI in severely burned patients remains beneficial, as it attenuates insulin resistance.

### 2.5. Angiotensin II Receptor Blockers

Angiotensin II receptor blockers (ARBs) (candesartan cilexetil, eprosartan, irbesartan, losartan, olmesartan medoxomil, telmisartan, valsartan, etc.) generally have similar indications as ACEI, but not identical [77,78,79]. Their mechanism of action is different: ARBs bind and block the angiotensin receptors, mainly AT1 receptor (angiotensin II receptor type 1), and ARBs stimulate the release of renin, with a consequent increase in AngII (angiotensin II) plasmatic levels [77]. Circulating AngII stimulates the AT2 receptor (angiotensin II receptor type 2). Given that the AT1 receptors are blocked, angiotensin II cannot exert its actions.

An important metabolic consequence of ARB administration consists of increasing insulin sensitivity through the activation of peroxisome proliferator-activated receptors γ (PPARγ) [80], and telmisartan seems to be the most effective [81,82]. Peroxisome proliferator-activated receptors (PPARs) are nuclear receptors activated by intracellular lipids (key metabolic ligands) and regulate the transcription of genes controlling lipid homeostasis (the differentiation of adipocytes and lipid metabolism) [83]. There are three PPAR subtypes (α, γ, and δ), which are unevenly distributed in different tissues. PPARγ regulate the flux of cholesterol into and out of the cell, lipid storage, and fat cell differentiation [83].

When compared with the PPARγ ligand pioglitazone, ARBs behave like partial PPARγ agonists. Pioglitazone is well known as a major antidiabetic agent. The activation of PPARγ and PPARα by pioglitazone modulates the transcription of genes with a role in the regulation of glucose metabolism and lipid metabolism in the liver, fat tissue, and muscles. This action results in a reduction in insulin resistance in peripheral tissues and the liver [84].

In animal models, irbesartan improves insulin sensitivity due to the important upregulation of the PPARγ target gene GLUT4 [85,86]. Accumulated data demonstrate that the reduction of insulin resistance by some ARBs is linked to the activation of PPARγ and not to blockade of AT1 receptors [87].

To the best of our knowledge, there are no clinical studies related to the beneficial effects of ARBs on insulin resistance in severely burned patients, but we may speculate about their usefulness in the modulation of the hyperglycemic status after severe burns.

### 2.6. Statins and Insulin Resistance in Burns

**Figure 4:**
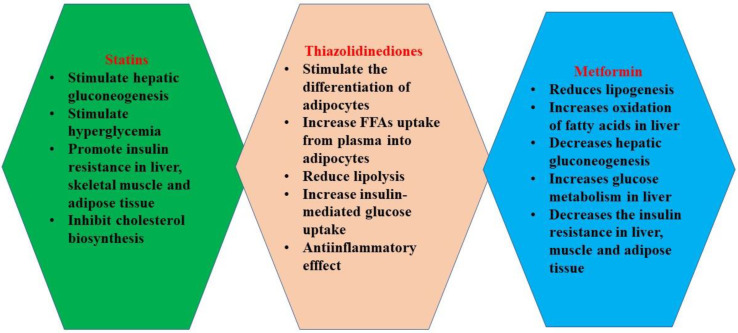
Effects of statins, thiazolidinediones, and metformin.

Statins are used in the treatment of dyslipidemia [88] and represent first-line drugs administered for the prevention of primary and secondary cardiovascular diseases [89,90]. By inhibiting the conversion of HMG-CoA (3-hydroxy-3-methyl-glutaryl-coenzyme A) to mevalonic acid, they are HMG-CoA reductase inhibitors [91]; HMG-CoA reductase is the rate-determining enzyme in the biosynthesis of cholesterol [91]. Statins determine a decrease in cholesterol synthesis (Figure 4), so there is a decrease in intracellular cholesterol levels, triggering increased expression of LDL-R (low-density lipoprotein receptor) in the liver and peripheral tissues. LDL-R binds LDL-C and removes it from circulation, leading to decreased blood levels of LDL-C [92].

Observational studies, randomized clinical studies, and meta-analyses have revealed that long-term treatment with statins increases the risk of developing type 2 diabetes mellitus [93]. However, the risk was not the same for all members of the statins group [94].

The diabetogenic effect of statins is explained through several mechanisms of action that coexist:

- Increase in hepatocyte gluconeogenesis through upregulation of the expression of genes codifying the synthesis of nodal enzymes [95];
- Decrease in glucose uptake in peripheral tissues by impairing the insulin signaling pathway and downregulation of GLUT4 with the development of insulin resistance [96,97];
- Decrease in insulin production due to β-insular cell damage [98];
- Accumulation of FFAs in hepatocytes [93];
- Decrease in the production of adiponectin and leptin by adipose tissue [99];
- Alteration of the expression profile of microRNAs involved in the regulation of glucose metabolism and lipid metabolism [100];
- Induction of insulin resistance [101].

The induction of insulin resistance by statins determines an abnormally reduced response of insulin-sensitive cells to normal levels of circulating insulin, as noticed in hepatocytes, adipocytes, and myocytes [101]. It appears that statins interfere with the insulin signaling pathway at different levels, in a tissue-dependent manner [90].

#### 2.6.1. Statins and Hepatocyte Insulin Sensitivity

GLUT2 (glucose transporter 2) is the main transporter for glucose in the kidneys, small intestine mucosa, β-pancreatic cells, and liver [102], and it works together with GK (glucokinase). Although GLUT2 has low affinity for glucose, its transport capacity exceeds GK’s phosphorylation capacity. Consequently, glucose phosphorylation is a rate-limiting process for glucose uptake in hepatocytes [102].

Animal studies revealed that atorvastatin, simvastatin, pitavastatin, and lovastatin markedly reduced glucose utilization in hepatocytes, leading to postprandial hyperglycemia [103]. The same animal studies showed that atorvastatin downregulated the expression of GLUT2 and GK in the liver, resulting in postprandial reduced uptake of glucose into hepatocytes [103], with consequent hyperglycemia.

Statins stimulate hepatic gluconeogenesis through upregulation of the expression of genes encoding key-limiting gluconeogenetic enzymes, G6P-ase (glucose-6-phosphatase) and PEPCK (phosphoenolpyruvate carboxykinase) [104,105], further contributing to hyperglycemia, which is a marker of insulin resistance.

Atorvastatin and rosuvastatin upregulate thyroid hormone responsive spot 14 protein (THRSP) and, consequently, induce insulin resistance in the liver [101]. THRSP is a nuclear protein that is involved in the control of lipid metabolism. Its transcription is induced by thyroid hormones (and other factors: glucose, insulin) and inhibited by glucagon and conjugated linoleic acid [106]. It has been identified in the liver, adipose tissue, and mammary glands [107,108], and it controls the expression of lipogenic genes [109,110,111].

The upregulation of lipogenic genes results in the accumulation of FFAs in the liver, an indirect marker of insulin resistance [112] and the development of type 2 diabetes mellitus [113].

#### 2.6.2. Statins and Skeletal Muscle Insulin Sensitivity

The skeletal muscle is the most prominent consumer of circulating glucose [114,115], and any decrease in the insulin sensitivity of the skeletal muscle determines hyperglycemia and accentuates the hypercatabolic state of a severely burned patient [116].

Atorvastatin and simvastatin induce insulin resistance in skeletal muscles [117,118] through multiple mechanisms:

- It appears that simvastatin impairs the phosphorylation of mTOR (mammalian target of rapamycin), which prevents the phosphorylation of AKT/PKB (protein kinase B) at Ser473 [119]; this results in a lack of activation of GSK3β, thus determining an impairment of GLUT4 translocation to the skeletal muscles’ cell membranes [117,119], finally decreasing glucose uptake by the cells.
- Simvastatin inhibits the translocation of GLUT4 to the plasma membrane through inhibition of prenylation of RabGTPases [120].

GTPases (guanosine triphosphatases) are enzymes that hydrolyze guanosine triphosphate to guanosine diphosphate, “working as molecular switches in the regulation of cell responses to extracellular signals” [121], and are involved in many processes, including transmembrane transportation of proteins and transport of vesicles within the cell [121].

RabGTPases are a family of small GTPases that control the membrane trafficking [122]. In order to be active, they need to be prenylated or double prenylated by GEFs (guanine nucleotide exchange factors) [123]. Simvastatin inhibits the prenylation of RabGTPases, thus altering the translocation of GLUT4 to the cell membrane and, consequently, decreasing glucose uptake [120].

c. Statins inhibit the biosynthesis of cholesterol [91], acting as hypolipemiant drugs [91]. By decreasing intracellular cholesterol, Atorvastatin decreases the translocation of GLUT4 [124], a partially cholesterol-dependent process [125]. The result is a decrease in glucose uptake in the skeletal muscle.
d. It has been demonstrated that the accumulation of FFAs in the skeletal muscle inhibits glucose uptake through inhibition of glucose transport and glucose phosphorylation [126]. FFAs in excess compete with glucose oxidation by decreasing glucose-6-phosphate formation [126], which is the form that permits glucose to be metabolized in the cell.

The blocking of HMG CoA reductase by simvastatin [91] determines the intracellular build-up of Acetyl CoA, with consequent upregulation of fatty acid synthesis and accumulation of FFAs in muscle cells [118], promoting insulin resistance.

#### 2.6.3. Statins and Adipocyte Insulin Sensitivity

Statins decrease adipocyte insulin sensitivity through several mechanisms:

- Statins inhibit the insulin-induced translocation of GLUT4 [127]. By inhibiting isoprenoid synthesis [96], statins impede on the active membrane fraction of Rab4 GTPase and RhoA (small GTPases involved in transmembrane transport) [127], because their active forms are dependent on isoprenilation [128]. Therefore, the insulin-stimulated translocation of GLUT4 is decreased [127], which results in reduced glucose uptake in the adipocytes.
- Statins impede caveolae formation [129].

After translocation from the cytosol (induced by insulin), GLUT4 docks on specific plasma membrane domains called caveolae [130]. The normal conformation and dynamics of caveolae are highly dependent on cholesterol [129] and are regulated by special proteins (cavin and caveolin) [129,131]. Caveolin-1 is a molecular stabilizer of the insulin receptor in adipocytes, being a chaperone for normal function of the insulin signaling pathway [132]. By inhibiting the synthesis of cholesterol, statins alter the formation and dynamics of caveolae, with secondary insulin resistance [129].

c. Statins indirectly reduce the secretion of high-molecular-weight oligomers of adiponectin, resulting in insulin resistance [133].

Adiponectin has an insulin sensitizing effect through binding to its receptors in adipocytes, AdipoR1 and AdipoR2; this results in activation of the AMPK (AMP-activated protein kinase) and PPARα (peroxisome proliferator-activated receptors α) signaling pathways [134].

Adiponectin stimulates the activity of LPL (lipoprotein lipase) with consequent increased uptake of TAG (triglyceride) in ectopic white adipose tissue in the liver and muscle [135]; by upregulation of AMPK in skeletal muscle, adiponectin stimulates lipid oxidation in muscles [135]. Consequently, adiponectin reduces lipid-induced insulin resistance in the liver and in skeletal muscles. A reduction in adiponectin levels, secondary to statin administration [133], has been associated with insulin resistance [134].

We may conclude that statins, despite their beneficial effect in lowering circulating LDL and VLDL in dyslipidemic patients, have an important secondary effect: decreasing the insulin sensitivity of the liver, adipose tissue, and skeletal muscle. For this reason, long-term use of statins in patients who have survived major burns might not be beneficial.

### 2.7. Thiazolidinediones

Thiazolidinediones (rosiglitazone, pioglitazone) are insulin sensitizers [136]. They are ligands for PPAR-γ (peroxisome proliferator-activated receptors gamma), which are nuclear receptors that, once activated, control the transcription of genes involved in lipid [83] and glucose homeostasis [136]. These nuclear receptors are expressed in the adipose tissue, myocardium, skeletal muscle, liver, vascular smooth muscle, intestine, kidneys, and macrophages [137]. Their receptors are naturally ligated by small lipophilic molecules (arachidonic acid, oxidized linoleic acid). Their activation stimulates the differentiation of adipocytes, increases FFA uptake from plasma into adipocytes, reduces lipolysis, and increases insulin-mediated glucose uptake [83]. Glucose uptake is increased mainly in the adipose tissue, skeletal muscle, and liver [83,136].

They are traditionally used in the prevention and treatment of type 2 diabetes mellitus [137,138], but experimental studies proved that rosiglitazone prevents the deepening of burns [139] and also protects against burn-induced oxidative injury of remote organs through an anti-inflammatory effect [140]. It appears that the activation of PPAR-γ has an anti-inflammatory effect in smooth muscle cells from the blood vessels’ walls [141]. The regulatory effect upon glucose metabolism is progressively established 2–3 months from the beginning of the treatment [142].

The main concerns about thiazolidinediones are related to secondary adverse effects, which demand caution in their usage in severe burns:

- Liver toxicity: This was noticed in troglitazone, which has been withdrawn from the market [143];
- Fluid retention and edema: This developed in thiazolidinedione monotherapy, but especially in association with insulin therapy [144], and may precipitate congestive heart failure in diabetic patients [145];
- Increase in body adipose tissue: Thiazolidinediones increase the uptake of FFAs from blood into the adipocytes, diminish the lipid stores in extra-adipose tissues (liver, skeletal muscle), and augment lipid storage in adipose tissue (especially subcutaneous adipose tissue) [146];
- Myocardial ischemia: There have been concerns related to the use of thiazolidinediones and myocardial ischemia [147,148]. Other studies did not find any concluding link between the use of thiazolidinediones and heart ischemic events [149,150];
- Skeletal fractures: It appears that rosiglitazone and pioglitazone decrease bone density and increase the risk of fractures in diabetic patients [151,152,153,154].

### 2.8. Metformin

Metformin is a well-known biguanide antidiabetic drug that acts on multiple levels upon glucose and lipid metabolism: it reduces lipogenesis, increases the oxidation of fatty acids in liver, and decreases hepatic gluconeogenesis. It also increases glucose metabolism in the liver [155]. It is well documented that metformin decreases hepatic insulin resistance through several mechanisms: it reduces AMP (adenosine monophosphate) [156], augments insulin inhibition upon gluconeogenesis in hepatocytes [156,157], and suppresses hepatic glucagon signaling [156,157,158].

Its action of augmenting insulin sensitivity in peripheral tissues was initially doubted: some authors considered that metformin acts predominantly upon the liver and has little effect upon skeletal muscle, which is the main glucose consumer [159]. During recent years, it has been proven that metformin decreases insulin resistance in the liver, muscle, and adipose tissue, too [160,161,162,163,164].

In severely burned patients, metformin has a very good effect of lowering plasmatic glucose levels, comparable with insulin administration [164]. The principal mechanism of action is well demonstrated in the liver, consisting of activation of AMPK, but is not clear for the decrease in insulin resistance in adipose tissues and skeletal muscles [157].

Due to its multiple favorable effects, including the increase in insulin sensitivity and the mitigation of hyperglycemia, metformin is currently used for pharmacologic modulation of the hyperglycemic–hypermetabolic state that develops in severe burns [165].

Table 1 summarizes the main effects of the drugs described in this review article.

## 3. Conclusions

Patients with severe burns, apart from a systemic inflammatory response, develop insulin resistance as part of the hypermetabolic state associated with the trauma. This state is hallmarked by hyperglycemia, augmented hepatic gluconeogenesis, decreased glucose uptake in skeletal muscle and adipose tissue, increased plasmatic levels of free fatty acids, and increased proteolysis in skeletal muscles. Therefore, managing insulin resistance is a priority in the therapeutic approach for these patients.

These patients need intensive care support and surgical treatment. Many of them have chronic medications for associated health conditions that precede the acute event (the burn trauma), and they receive several new drugs as part of the complex treatment plan for the severe burn. We must keep in mind that some of the pre-existing medications or some of the new administered drugs might interfere with the insulin sensitivity of the liver, brain, skeletal muscle, adipose tissue, etc. Therefore, we must take special caution in the administration of drugs that have been shown to increase insulin resistance and to accentuate the hypermetabolic state, thereby worsening the prognosis of the patient, such as: statins, vitamin B3, and second-generation antipsychotic drugs. On the other hand, β blockers, angiotensin-converting enzyme inhibitors, angiotensin II receptor blockers, thiazolidinediones, and metformin are proven to increase insulin sensitivity and may play a beneficial role in the therapeutic approach to severely burned patients.

Moreover, the drugs presented in this review have different action mechanisms and, subsequently, have distinct effects. Future studies are needed in order to evaluate their complimentary results, as the association of several types of medication could provide a better approach to insulin resistance. Reducing hyperglycemia and enhancing insulin sensitivity are priorities for the successful treatment of these patients.

## Figures and Tables

**Table 1:** Drugs that might interfere with insulin resistance in severe burns.

Drugs	Cardiac Effects	Blood Pressure	Hypermetabolism	Insulin Resistance	Lipid Metabolism	Catecholamines
β blockers	cardio protective effects/decrease tachycardia and workload	↓ hypertension	↓ hypercatabolic state ↓ hyperglycemia	↓	inhibit lipolysis ↓ FFAs, ↓ cholesterol, ↓ TG levels	-
Antipsychotics	-	-	↑ hyperglycemia	↑	stimulate lipolysis ↑ FFAs, ↑ cholesterol, ↑ TG levels	↑ Plasmatic levels of catecholamines
Vitamin B3	-	-	↑ hyperglycemia	↑	stimulate lipolysis ↑ FFAs, ↑ cholesterol, ↑ TG levels	↑ plasmatic levels of catecholamines
ACEI	cardio protective effects	↓ hypertension	-	↓	ameliorate lipid profile	↓ plasmatic levels of catecholamines
ARBs	cardio protective effects	↓ hypertension	-	↓	ameliorate lipid profile	
Statins	-	-	↑ hypercatabolic state ↑ hyperglycemia	↑	↓ cholesterol levels ↑ accumulation of FFAs in muscles and the liver ↓ LDL, VLDL	-
Thiazolidinediones	might be linked to myocardial ischemia	might precipitate congestive heart failure in diabetic patients	↓ hyperglycemia	↓	↑ FFA accumulation in adipose tissue	-
Metformin	-	-	↓ hypercatabolic state ↓ hyperglycemia	↓	↑ oxidation of FFA in the liver reduces lipogenesis	-

In the columns of insulin resistance, respectively lipid metabolism: The black arrow points to the effects upon insulin resistance. The blue arrow shows the effects upon lipid metabolism.
